# Effects of obesity on CC16 and their potential role in overweight/obese asthma

**DOI:** 10.1186/s12931-022-02038-1

**Published:** 2022-06-29

**Authors:** Houman Goudarzi, Hirokazu Kimura, Hiroki Kimura, Hironi Makita, Munehiro Matsumoto, Nozomu Takei, Kaoruko Shimizu, Masaru Suzuki, Taku Watanabe, Eiki Kikuchi, Hiroshi Ohira, Ichizo Tsujino, Jun Sakakibara-Konishi, Naofumi Shinagawa, Noriharu Shijubo, Hirokazu Sato, Katsunori Shigehara, Kichizo Kaga, Yasuhiro Hida, Soichi Murakami, Yuma Ebihara, Akinobu Nakamura, Hideaki Miyoshi, Satoshi Hirano, Nobuyuki Hizawa, Tatsuya Atsumi, Shau-ku Huang, Yoichi M. Ito, Masaharu Nishimura, Satoshi Konno

**Affiliations:** 1 Department of Respiratory Medicine, Faculty of Medicine and Graduate School of Medicine, Hokkaido University, North 15 West 7, Kita-Ku, Sapporo, 060-8638 Japan; 2 Department of Respiratory Medicine, JR Sapporo Hospital, Sapporo, Japan; 3 Department of Health Management, JR Sapporo Hospital, Sapporo, Japan; 4 Department of Human Immunology, Research Institute for Frontier Medicine, Sapporo Medical University School of Medicine, Sapporo, Japan; 5 Ai Medical Clinic, Sapporo, Japan; 6 Department of Cardiovascular and Thoracic Surgery, Faculty of Medicine, Hokkaido University, Sapporo, Japan; 7 Department of Gastroenterological Surgery II, Faculty of Medicine and Graduate School of Medicine, Hokkaido University, Sapporo, Japan; 8 Department of Rheumatology Endocrinology and Nephrology, Faculty of Medicine and Graduate School of Medicine, Hokkaido University, Sapporo, Japan; 9 Division of Diabetes and Obesity, Faculty of Medicine and Graduate School of Medicine, Hokkaido University, Sapporo, Japan; 10 Division of Respiratory Medicine, Institute of Clinical Medicine, University of Tsukuba, Ibaraki, Japan; 11 National Institute of Environmental Health Sciences, National Health Research Institutes, Miaoli, Taiwan; 12 Johns Hopkins University School of Medicine, Baltimore, MD USA; 13 Biostatics Division, Clinical Research and Medical Innovation Center, Hokkaido University Hospital, Sapporo, Japan

**Keywords:** CC16, Overweight and obesity, BMI, Asthma, Clinical asthma measures

## Abstract

**Introduction:**

Club cell secretory protein-16 (CC16) is a major anti-inflammatory protein expressed in the airway; however, the potential role of CC16 on overweight/obese asthma has not been assessed. In this study, we examined whether obesity reduces airway/circulatory CC16 levels using experimental and epidemiological studies. Then, we explored the mediatory role of CC16 in the relationship of overweight/obesity with clinical asthma measures.

**Methods:**

Circulating CC16 levels were assessed by ELISA in three independent human populations, including two groups of healthy and general populations and asthma patients. The percentage of cells expressing club markers in obese vs. non-obese mice and human airways was determined by immunohistochemistry. A causal mediation analysis was conducted to determine whether circulatory CC16 acted as a mediator between overweight/obesity and clinical asthma measures.

**Results:**

BMI was significantly and monotonously associated with reduced circulating CC16 levels in all populations. The percentage of CC16-expressing cells was reduced in the small airways of both mice and humans with obesity. Finally, mediation analysis revealed significant contributions of circulatory CC16 in the association between BMI and clinical asthma measures; 21.8% of its total effect in BMI’s association with airway hyperresponsiveness of healthy subjects (p = 0.09), 26.4% with asthma severity (p = 0.030), and 23% with the required dose of inhaled corticosteroid (p = 0.042). In logistic regression analysis, 1-SD decrease in serum CC16 levels of asthma patients was associated with 87% increased odds for high dose ICS requirement (p < 0.001).

**Conclusions:**

We demonstrate that airway/circulating CC16, which is inversely associated with BMI, may mediate development and severity in overweight/obese asthma.

**Supplementary Information:**

The online version contains supplementary material available at 10.1186/s12931-022-02038-1.

## Introduction

Epidemiologic studies have consistently demonstrated that obesity is associated with the prevalence of asthma [1–7], as well as poor disease control and increased severity [8–10]. Several mechanisms have been postulated to explain how obesity affects the severity of asthma, including abnormal respiratory mechanics [11], bronchial hyper-responsiveness [12], steroid resistance [13–15], and an increased prevalence of obesity-associated inflammation and comorbidities [10, 16–18]. However, the molecular basis for the obesity–asthma association remains unclear.

Club cell secretory protein-16 (CC16), also known as secretoglobin 1A1 (SCGB1A1) or CC10, is expressed primarily in the respiratory tract and is a potent anti-inflammatory agent that protects the airway from inflammation [19]. Mice deficient in CC16 exhibit higher levels of allergic airway inflammation [20], and the reconstitution of CC16 in CC16-deficient mice can reverse this [21]. In humans, studies have shown significant associations between decreased blood CC16 levels and asthma outcomes, such as airway hyperresponsiveness (AHR), nocturnal asthma, and steroid resistance [22–24]. CC16 is primarily produced in the respiratory tract and it has been proposed to be a clinically useful serum biomarker for the management of chronic airway diseases, such as chronic obstructive pulmonary disease (COPD), and asthma [22, 24, 25].

Given that CC16 is a major anti-inflammatory protein in the airway, coupled with the relationship between CC16 and asthma, we hypothesized that it is involved in the pathogenesis of obese asthma. In our preliminary data analysis, we found an inverse correlation between BMI and serum CC16 in patients with asthma. For an extension of the potential effects of obesity on CC16 levels in populations without asthma, we also examined two independent non-asthmatic populations to understand the association between BMI and circulating CC16 levels. With laboratory studies, we assessed the causal relationship between obesity and reduced CC16 levels. Finally, using mediation analysis, we examined to what extent CC16 could be involved in the effect of BMI on asthma severity, as well as AHR, in healthy subjects.

## Materials and methods

### Study populations

We examined circulatory CC16 in three independent populations as follows: (1) participants undergoing annual health check-ups (Population 1, n = 357): Japanese participants at the JR Sapporo Hospital (Sapporo, Japan) between 2009 and 2010, requested to answer questionnaires, including the European Community Respiratory Health Survey (ECRHS) questionnaire [26, 27]; (2) young healthy participants (Population 2, n = 137): asymptomatic individuals with no history of wheezing, shortness of breath, or other respiratory diseases, and none reported any recent respiratory infections based on written questionnaires and direct questioning (see Additional file 1) [28]; (3) patients with asthma (Population 3, n = 206): the recruitment of patients with asthma has been previously described [29, 30], and the diagnosis of severe asthma was based on the American Thoracic Society (ATS) criteria for refractory asthma in 2000 [31], with slight modifications [30]. This study was approved by the ethics committees of the Research Review Board of Hokkaido University (for Populations 2 and 3; 14-057, 15-041), Hokkaido University Hospital (009-0205, 017-0258, 018-0091), and JR Sapporo hospital (Population 1; 2012-5, 14-057). Written informed consent was obtained directly from all participants.

### Influence of the CC16 A38G polymorphism on circulating CC16 levels in all populations

Additional file 1: Table S1 and Figure S1 show the allele frequency and serum/plasma CC16 levels according to the A38G genotype. The frequency of this polymorphism did not deviate significantly from the Hardy–Weinberg equilibrium (p > 0.05) in Populations 1, 2, and 3. In all populations, CC16 levels differed significantly and in a monotonic manner based on genotype. Participants with the AA genotype had the lowest circulating CC16 levels. Therefore, we included *CC16* polymorphisms in our adjusted models.

### Nonspecific airway responsiveness in young healthy participants (Population 2)

For population 2, we performed the methacholine-induced bronchoconstriction test. Nonspecific airway responsiveness, as indicated by the degree of methacholine-induced bronchoconstriction, was determined by continuous methacholine inhalation with simultaneous measurement of respiratory resistance (Astograph; Chest, Tokyo, Japan), as described previously [23, 28]. After recording baseline respiratory resistance, beginning with the lowest concentration, increasing doses of methacholine were inhaled at 1-min intervals. The cumulative dose values of inhaled methacholine, measured at the inflection point at which respiratory conductance started to decrease (Dmin), were used as an index of AHR. One Dmin unit represented a 1-min inhalation of 1 mg/mL of methacholine (for more details, see Additional file 1).

### Lean vs. obese mice

C57BL/6J female mice were randomly divided into two groups fed a normal or high-fat diet for 6 weeks (see Additional file 1).

### Measurement of biomarkers

Serum or plasma samples were stored at − 80 °C until assayed for CC16. Serum or plasma CC16 levels were measured using a commercial enzyme-linked immunosorbent assay (ELISA) kit (Bio Vendor Laboratory Medicine, Inc., Brno, Czech Republic), as reported in previous studies [23]. For details of surfactant protein (SP)-A, and SP-D measurements in mice, see Additional file 1.

### Mouse and human lung tissue samples for immunohistochemistry

Formalin-fixed cancer-free lung samples of lung cancer cases who were never-smokers without any respiratory diseases were immunostained for CC16, SCGB3A2, and MUC5AC. For more details see Additional file 1: Fig. S2 and Table S2).

### Statistical analysis

To compare differences within and between groups, we used Student’s *t*-tests, and one-way analysis of variance for parametric continuous variables, a Mann–Whitney U test for non-parametric continuous variables, and a chi-square test for categorical variables. To assess the association of BMI with circulatory CC16 cross-sectionally, we divided BMI into three groups, and least-square means and 95% confidence intervals (CIs) for circulatory CC16 were calculated. To calculate the trend in p-values, we used linear contrast coefficients, as reported previously [4, 10]. We divided budesonide equivalent dose into low (200–400), medium (> 400–800), and high (> 800) dose groups in asthma patients (population 3) according to Global Initiative for Asthma (GINA) guideline [32] and examined the associations between serum CC16 levels and the risk of high dose of ICS requirement using logistic regression analyses. We selected confounders in the analysis based on available mechanistic and biological knowledge (see Additional file 1). Statistical analyses were performed using the statistical software package JMP version 13 (SAS Institute Inc., Cary, NC, USA). For all statistical analyses, p < 0.05 was considered significant.

### Mediation analysis

To estimate the extent to which circulatory CC16 explains the association between BMI and asthma-related outcomes, we conducted a causal mediation analysis, based on a counterfactual approach using the CAUSALMED procedure and estimated the total effect as the sum of direct and indirect effects [33, 34]. The indirect effect referred to the effect of BMI on clinical asthma measures through circulatory CC16 as a mediator. The direct effect referred to the remaining effect that is not through the mediator. The mediation analysis was conducted with SAS 9.4 (SAS Institute, Cary, NC).

## Results

### Participant characteristics

In Population 1 who had their annual health check-up (Additional file 1: Table S3), 43.1% of participants were females. The mean ± standard deviation (SD) age and BMI were 50.5 ± 7.7 years and 22.7 ± 3.1 kg/m^2^, respectively. Current smokers (vs. non-current smokers) and female (vs. male) participants had significantly lower levels of serum CC16 (Additional file 1: Table S4). In Population 2 (young healthy participants), 29.2% were female. Only 10.2% of the participants were current smokers, the mean ± SD for age and BMI were 24.0 ± 3.4 years and 21.6 ± 2.6 kg/m^2^, respectively (Additional file 1: Tables S5 and S6). In Population 3 (asthma patients), the mean ± SD for age and BMI were 59.5 ± 13.8 years and 24.9 ± 5.0 kg/m^2^, respectively (Additional file 1: Table S7). Further, 59.7% of the participants were female and 127 (61.7%) had severe asthma. The number of patients who were overweight/obese (BMI ≥ 25 kg/m^2^) was significantly higher in patients with severe asthma than in those with non-severe asthma (50.4% vs. 32.9%, Additional file 1: Table S8). The serum CC16 levels in patients with asthma were significantly lower in women than in men [geometric means (GMs), 6.3 vs. 7.7 ng/mL] and in patients with severe asthma than in those with non-severe asthma (GM, 6.1 vs. 8.2 ng/mL; Additional file 1: Table S9).

### BMI is inversely associated with circulating CC16 levels in a dose–response manner

**Fig. 1:**
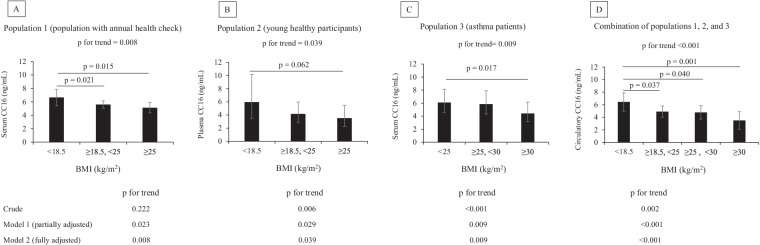
Association of circulating CC16 levels and categorical BMI in three populations. The values are shown as the least-square means, and the error bars depict the upper and lower 95% CI. Figures indicate fully adjusted associations between BMI values and circulatory CC16 levels (ng/mL). Model 1 (partially adjusted model): adjusted by age, sex, smoking status, and history of doctor-diagnosed asthma for population 1; adjusted by sex, age, smoking status for population 2; adjusted by age, sex, smoking status, and asthma severity for population 3; for the combination of populations 1, 2, and 3; adjusted by age, sex, and smoking status. Model 2 (fully adjusted model): adjusted for covariates in Model 1 and the *CC16* A38G polymorphism (rs3741240)

We examined the association between circulating CC16 levels and BMI in all populations using crude and adjusted models (Fig. 1A–C). We found a trend for a significant decline in CC16 levels across BMI tertiles in Populations 1 (p for the trend = 0.008) and 2 (p for the trend = 0 0.039) in fully adjusted models. We found the same results in asthma patients (p for the trend = 0.009; Fig. 1C). Finally, a combination of these three populations showed the same results (Fig. 1D) and the exclusion of current smokers did not alter the results (p for the trend < 0.001; Additional file 1: Fig. S3).

### Comparison of CC16-, SCGB3A2-, and MUC5AC-expressing cells in the small airways

**Fig. 2:**
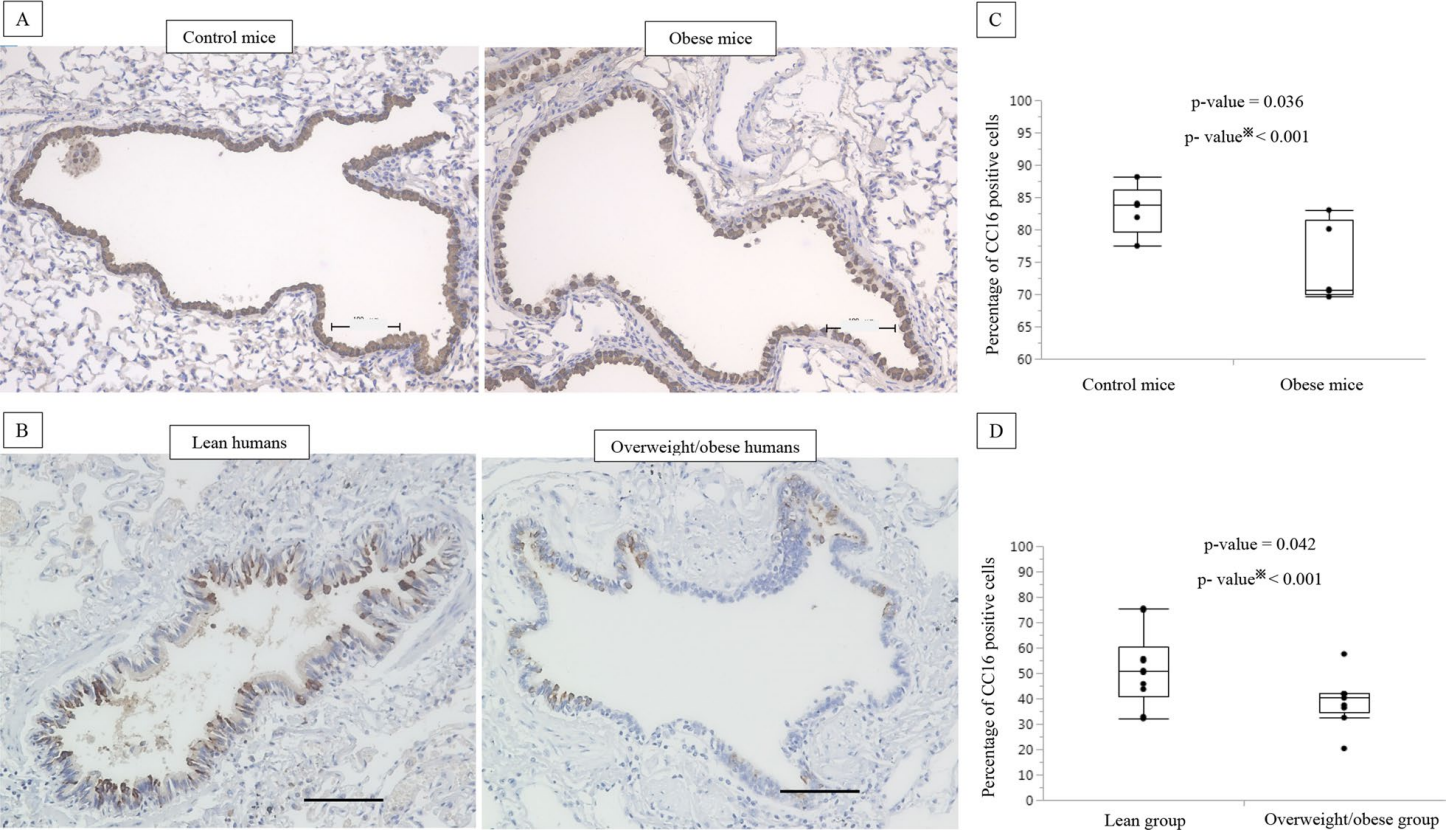
Decreased number of CC16 expressing cells in small airways of obese mice and overweight/obese humans. Representative photomicrographs of CC16 immuno-histochemical staining of obese vs. control mice (n = 5 mice per group) (**A**) and overweight/obese vs. lean human airways (**B**). Comparison of the percentage of CC16 expressing cells in the small airways of obese vs. normal control mice (n = 5 mice per group) (**C**). Comparison of the percentage of CC16 expressing cells in the small airways of overweight/obese vs. lean humans (n = 10 for the lean group, and n = 9 for the overweight/obese group) (**D**). The number of cells expressing each target protein in the small airways was manually and randomly counted in five randomly selected fields by two blinded examiners to determine the average percentage of positive cells/sample. The scale bars are 100 µm. ※split-plot method (ANOVA)

Using immunohistochemistry, we observed a significant decrease in CC16-expressing small airway epithelial cells in obese mice compared to that in control mice (p = 0.036; Fig. 2A, C). We also found that humans who were overweight/obese had a lower number of CC16-expressing cells compared to that in lean individuals (p = 0.042; Fig. 2B, D, Additional file 1: Table S2). Additionally, we examined the number of SCGB3A2 (another biomarker of club cells)-expressing cells (Additional file 1: Fig. S4) and found reduced numbers in the airways of humans and mice with obesity compared to those in controls without obesity. We performed periodic acid–Schiff and MUC5AC staining in human and mouse samples; however, no significant differences were found in the percentages of mucin-producing cells between subjects with and without obesity (data not shown).

### Obesity reduces circulatory CC16 levels but not SP-A and SP-D levels

Serum CC16 levels were significantly lower in obese mice than in non-obese control mice (p = 0.002). However, the serum levels of SP-A and SP-D, other products of club cells, did not vary between obese and non-obese control mice (Additional file 1: Fig. S5). We also found no significant difference in *scgb1a1* gene expression (encodes CC16 protein) in the lung of obese vs lean mice (data not shown).

### Association between BMI and airway hyperresponsiveness mediated by CC16 among young healthy participants (Population 2)

**Fig. 3:**
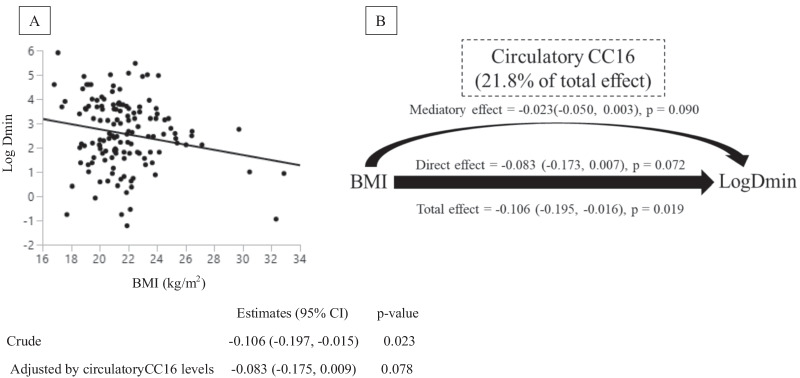
BMI, circulatory CC16, and airway hyperresponsiveness in young healthy participants (Population 2). **A** The figure indicates the unadjusted associations between BMI and Log Dmin. **B** Mediation analyses were performed to estimate the direct and mediation effect of BMI on Log Dmin (airway hyperresponsiveness; AHR) mediated by circulatory CC16 in young healthy participants. Numbers in the parentheses indicate the estimates, 95% confidence intervals (in the parentheses), and p-values

We found a significant inverse association of BMI with Log Dmin between values (p = 0.022), which was attenuated after adjusting for CC16 levels (p = 0.078; Fig. 3A). We also found a significant positive association between CC16 levels and log Dmin in crude and adjusted models. Of note, this association remained significant after controlling for BMI (p = 0.045; Additional file 1: Fig. S6). Therefore, we conducted a mediation analysis to access the potential mediatory role of CC16 in the association between BMI and AHR and found a mediating effect of CC16 on the BMI–AHR relationship accounting for 21.8% of the total effect, albeit non-significant (p = 0.09) (Fig. 3B).

### Association between BMI and asthma severity or requirement dose of inhaled corticosteroid (ICS) mediated by CC16 among patients with asthma (Population 3)

**Fig. 4:**
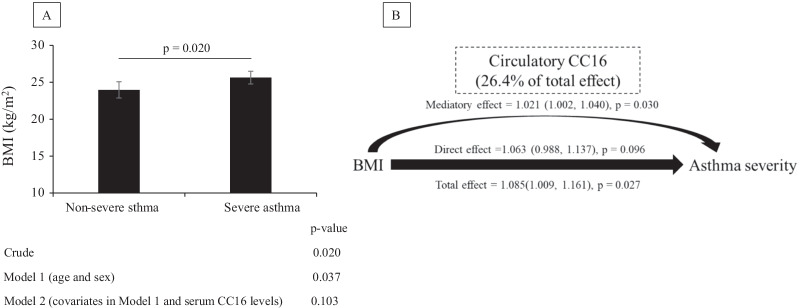
Association among, BMI, circulatory CC16, and asthma severity in patients with asthma (Population 3). **A** The values are shown as the least square means (95% confidence interval; CI). The figure shows the fully adjusted associations between asthma severity and circulatory CC16 levels (ng/mL; model 2). **B** Mediation analyses were performed to estimate the direct and mediation effect of BMI on asthma severity mediated by circulatory CC16 in asthma patients. Numbers indicate the odds ratio, 95% CIs (in parentheses), and p-values

BMI was significantly higher in patients with severe asthma than in those with non-severe asthma in the crude model (p = 0.020) and after adjustments for sex and age (p = 0.037); however, this association was attenuated after controlling for CC16 levels (p = 0.103; Fig. 4A). Of note, serum CC16 levels were significantly lower in patients with severe asthma than in those with non-severe asthma, even after controlling for BMI (p = 0.009; Additional file 1: Fig. S7). We, therefore, evaluated the potential mediatory effect of CC16 on the association between BMI and asthma severity. The results from the mediation analysis indicated a significant effect of BMI on asthma severity [odds ratio (OR): 1.085; 95% CI 1.009, 1.161; p = 0.027], whereas the CC16 mediatory effect was 26.4% of the total effect (OR: 1.021; 95% CI 1.002, 1.040; p = 0.030). Interestingly, the direct effects (not mediated through CC16) were not significant (OR: 1.063; 95% CI 0.998, 1.137; p = 0.096; Fig. 4B).

**Fig. 5:**
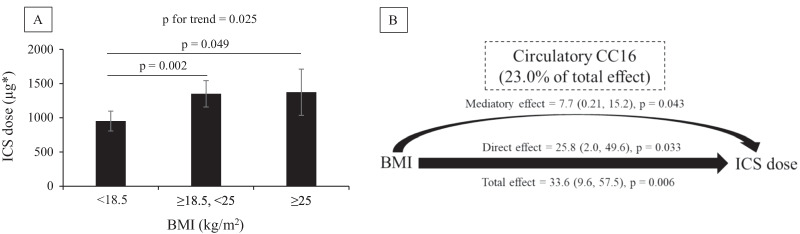
Association among BMI, circulatory CC16, and inhaled corticosteroid (ICS) dose in patients with asthma (Population 3). **A** The values are shown as the least square means (95% confidence interval; CI). Patients with current oral corticosteroid (OCS) use were excluded (n = 45). The figure shows the unadjusted association between BMI and ICS dose. **B** Mediation analyses were performed to estimate the direct and mediation effects of circulatory CC16 levels on the association between BMI and ICS dose in patients with asthma. Numbers indicate the estimates, 95% CIs (in the parentheses), and p-values

BMI was found to be positively associated with the required dose of ICS (p for trend = 0.025); however, this association was attenuated after adjusting for serum CC16 levels (p for trend = 0.101; Fig. 5A). Moreover, the significant association between CC16 levels and lower ICS dose did not change after controlling for BMI (Additional file 1: Fig. S8). We also found that CC16 mediated 23% of the total effect of BMI on requirement dose of ICS (coefficient: 7.7; 95% CI 0.21, 15.2; p = 0.043; Fig. 5B). We also divided ICS dose into low, medium, and high dose groups in asthma patients according to GINA guideline and examined the dose–response relationship between required ICS dose and circulatory CC16 (Additional file 1: Fig. S9) and found that the highest ICS dose group was associated with a − 3.55 ng/mL (95% CI − 5.35, − 1.74) in CC16 levels compared with the lowest ICS dose group (p for trend < 0.0001). We then tested the impact of CC16 change on the risk of high ICS dose (vs non-high ICS dose) requirement (Table 1) and found a 1-SD decrease in serum CC16 levels of asthma patients to be associated with 87% increased odds for high dose ICS requirement (95% CI 1.28–2.73; p < 0.001).

**Table 1:** Decreased serum CC16 levels and risk of high dose ICS requirement in asthma patients

Impact for a 1-SD decrease in serum CC16 levels	OR (95% CI)	p-value
Crude	1.87 (1.28, 2.73)	< 0.001
Model 1	1.88 (1.20, 2.83)	0.001
Model 2	1.75 (1.16, 2.65)	0.004

Patients with current oral corticosteroid use were excluded (n = 45). The figure indicates unadjusted associations between values of serum CC16 and ICS dose. Model 1 adjusted for age, sex, smoking status, pack-year, *CC16* A38G polymorphism (rs3741240). Model 2 adjusted for covariates in Model 1 and BMI

Equivalent to budesonide dose was used to divide asthma patients into high (n = 82) vs non-high groups (n = 79) according to the cutoff values provided by the GINA guideline

*OR* odds ratio, *CI* confidence interval

## Discussion

To the best of our knowledge, we have demonstrated for the first time that obesity is inversely associated with airway and circulatory CC16 levels. CC16 was significantly and inversely associated with being overweight/obese in asthmatic and non-asthmatic populations, regardless of smoking status and genetic polymorphisms. Obesity reduced circulating CC16 levels but not surfactant protein SP-A and SP-D levels in the mice in this study. Furthermore, we found fewer CC16-expressing cells in the airways of subjects with obesity. Whereas CC16 is expressed primarily in the respiratory tract, the inverse association between CC16 levels and BMI in participants without asthma suggests that high BMI itself could influence the CC16 levels, regardless of asthma. Based on the firm evidence regarding the causal relationship between obesity and reduced CC16 levels mentioned previously herein, we attempted, for the first time, to quantify the indirect effect of BMI on asthma outcomes that might be explained by CC16. Our results indicated evidence of CC16 effect on the relationship between obesity and examined asthma outcomes including AHR, asthma severity, and requirement dose of ICS, comprising 21.8%, 26.4%, and 23% of the total effect, respectively. Finally, a 1-SD decrease in serum CC16 levels was associated with around 80% increased risk of high ICS dose in asthma patients.

It has been shown that circulating CC16 levels are highly influenced by age. Guerra et al. [25] reported a U-shaped age distribution in CC16 levels among adults, which is in line with our results in Populations 1 and 3. In Population 2, we examined the association between circulating CC16 levels and BMI among young healthy participants within a narrow range of ages to minimize the influence of age and pulmonary diseases. Regarding smoking as another confounder [35], with a combination of the three human populations and excluding smokers, we showed that the association between BMI and CC16 levels is not confounded by smoking.

Notably, in Population 2, circulating CC16 levels and BMI were significantly associated with AHR in young healthy adults without asthma. Despite often being accompanied by respiratory symptoms, AHR can occur without any clinical respiratory symptoms, a condition termed asymptomatic AHR [36]. Although the clinical significance of asymptomatic AHR and its relationship with asthma is not fully understood, prospective studies have suggested that asymptomatic AHR is a risk factor for the development of future asthma in adults [37, 38]. We found that the association between BMI and AHR was attenuated after controlling for CC16 levels and the estimated mediation effect explained approximately 22% of the association between BMI and AHR. Although the p-value did not reach the threshold of statistical significance (p = 0.090), it could be because of the modest sample size of Population 2.

Among patients with asthma, BMI was inversely associated with serum CC16 levels, and patients with severe asthma had lower CC16 levels than did those with mild-to-moderate asthma. Furthermore, the significantly higher BMI seen in severe asthma patients compared to that in non-severe asthma patients was attenuated after adjusting for CC16 levels. Mediation analysis showed a significant indirect, but not direct, effect of BMI on asthma severity, suggesting an independent effect of BMI on asthma severity through CC16. Furthermore, the influence of BMI on the required dose of ICS was attenuated after controlling for CC16. Although an effect of high steroid doses on the suppression of CC16 expression could not be ruled out in this cross-sectional analysis, Chen et al. showed that the A allele of the *CC16* A38G genotype resulting in high CC16 levels is also associated with steroid sensitivity in children [24]. In line with this report, our results suggest that CC16 is involved in asthma severity, which is defined by steroid resistance [29, 38, 39].

In addition to *SCGB1A1* (which encodes CC16), *SCGB3A2* is another member of the *SCGB* gene superfamily expressed in club cells that plays an anti-inflammatory role in the lungs and is a marker of an early program of club cell fate [40, 41]. In lab studies, we found that obesity reduced the numbers of CC16- and SCGB3A2-expressing cells in the airways. This study, therefore, provides critical new insights into the mechanism by which obesity might affect club cell function and lineage, possibly by regulating secretory proteins in the airways. Because of the potential effects of obesity on epithelial cell differentiation, we also examined the number of mucin-expressing cells; however, we did not find any evidence supporting the role of obesity in the polarization of epithelial cell differentiation from club cells to goblet cells. Moreover, in contrast to that with serum CC16 levels, obesity did not reduce serum SP-A and SP-D levels, which can also be produced by type II pneumocytes, in addition to club cells.

To further clarify the influence of obesity on CC16 gene expression, we examined the mRNA expression of this gene in lung tissues of obese and control mice. However, we did not find a significant change in CC16 mRNA expression levels (data not shown). Obesity may therefore regulate CC16 protein levels post-transcriptionally. In addition, obesity may induce apoptosis in club cells resulting in a reduced number of CC16 expressing cells in the airway and a corresponding reduction in circulating CC16 levels.

Several reports have suggested that Asian populations with low levels of adiposity are more susceptible to the risk of non-communicable diseases than Western populations, including respiratory diseases [7, 42]. In a nationwide study, we examined the association between high BMI and asthma among Japanese adult participants aged 20 to 79 years, collecting data from a nationwide population-based survey of asthma prevalence in Japan (n = 22,962). The results suggested that Japanese people are likely to have asthma with a lesser degree of obesity (start at BMI 23.0) than Western populations. Further studies from Western populations are required to replicate our data, and the results may be more evident in such populations with a larger number of individuals with overweight and obesity. Furthermore, previous reports suggested high abdominal obesity, higher body fat percentage with lower body weight, and a tendency for abdominal obesity in Asian countries [43, 44], a risk factor for metabolic and respiratory diseases. Therefore, Asians may develop respiratory diseases with lower BMI.

The results of the current study are based on several strengths. We used three epidemiological studies to assess the association between BMI and CC16 levels in a dose–response manner creating several models to minimize potential residual confounding effects. We confirmed these findings from epidemiological studies with laboratory studies demonstrating a causal relationship between obesity and reduced CC16. However, the present study has several limitations. First, a cross-sectional analysis, particularly one examining associations among factors, such as BMI and CC16, in epidemiological studies, cannot determine causal relationships. Of note, we did observe that there was a significant inverse association between BMI and CC16 levels in three independent populations. In addition, CC16 is known to be expressed primarily in the respiratory tract, and club cells are the main source of circulating CC16. Our observation of a negative association between CC16 levels and BMI in participants without asthma strongly suggests that obesity itself can influence CC16 protein expression in the bronchioles. We also confirmed reduced circulating CC16 levels in obese mice, as well as fewer CC16-expressing airway epithelial cells in mice and humans with obesity, compared with those in the controls. Second, mediation analysis does not imply causation, and the results could be influenced by residual confounders. However, our results offer insight into the significant contribution of CC16 to the relationship between BMI and asthma-related outcomes. The results should therefore be interpreted with caution, and further studies are necessary to confirm the detailed pathophysiologic role of CC16 in the effect of obesity on asthma outcomes. Ultimately, we cannot rule out the effect of obesity on CC16 driven by dietary intake rather than by adipose tissue. Also, because of the lower prevalence of obesity in the general population and asthmatic patients in Japan compared to Western populations, it is worth replicating such data in western countries which have a significantly higher prevalence of obesity.

## Conclusions

In conclusion, we demonstrated that obesity is inversely associated with the level of a major anti-inflammatory pneumoprotein and immune modulator, CC16, in the airways and circulation. Being overweight/obese was inversely associated with the level of CC16 in asthmatic and non-asthmatic populations, and CC16 might be an important mediator of the association between BMI and asthma development and severity. Weight loss may improve respiratory outcomes in asthmatic and non-asthmatic obese populations through CC16-dependent mechanisms.

## Supplementary Information

**Additional file 1.** **Table S1.** Frequency of the *CC16* genetic polymorphism (rs3741240). **Table S2.** Characteristics of non-smoking patients with lung cancer without any pulmonary diseases by BMI group. **Table S3.** Characteristics of the participants who attended the hospital for routine health check-ups (population 1, n = 357). **Table S4.** Associations between characteristics and serum CC16 levels in participants who attended the hospital for routine health check-ups (population 1, n = 357). **Table S5.** Characteristics of the young healthy participants (population 2, n = 137). **Table S6.** Association between the characteristics of young healthy participants and plasma CC16 levels (population 2, n = 137). **Table S7.** Characteristics of patients with asthma (population 3, n = 206). **Table S8.** BMI distribution in patients with severe and non-severe asthma (population 3, n = 206). **Table S9.** Associations between characteristics and serum CC16 levels in patients with asthma (population 3, n = 206). **Fig. S1.** Circulating CC16 levels based on the *CC16* genetic polymorphism (rs3741240). **Fig. S2.** Flowchart for conducting CC16, SCBB3A2, and MUC5AC immunohistochemistry in the cancer-free lung tissues of never smoking human participants. **Fig. S3.** Association of circulating CC16 levels and categorical BMI in the combination of three populations excluding current smokers. **Fig. S4.** Decreased SCGB3A2 expressing cells in the airways of obese mice and overweight/obese humans. **Fig. S5.** Comparison of serum CC16 levels and blood SP-A and SP-D levels in obese vs. control mice. **Fig. S6.** Association of circulatory CC16 with airway hyperresponsiveness in young healthy participants (population 2). **Fig. S7.** Reduced serum CC16 levels among severe compared to non-severe asthma patients (population 3). **Fig. S8.** Inverse association of circulatory CC16 and inhaled corticosteroid (ICS) dose in asthma patients (population 3). **Fig. S9.** Circulatory CC16 levels according to required ICS dose in asthma patients (population 3).

## Data Availability

The datasets used and/or analysed during the current study are available from the corresponding author on reasonable request.

## Abbreviations

AHR: Airway hyperresponsiveness
ATS: American Thoracic Society
CC16: Club cell secretory protein-16
ECRHS: European Community Respiratory Health Survey
ELISA: Enzyme-linked immunosorbent assay
GINA: Global Initiative for Asthma
ICS: Inhaled corticosteroid
SP-A: Surfactant protein-A
SP-D: Surfactant protein-A

## References

[1] Beuther DA, Weiss ST, Sutherland ER (2006). Obesity and asthma. Am J Respir Crit Care Med.

[2] Shore SA (2008). Obesity and asthma: possible mechanisms. J Allergy Clin Immunol.

[3] Lugogo NL, Kraft M, Dixon AE (2010). Does obesity produce a distinct asthma phenotype?. J Appl Physiol.

[4] Goudarzi H, Konno S, Araki A, Miyashita C, Itoh S, Ait Bamai Y (2018). Contrasting associations of maternal smoking and pre-pregnancy BMI with wheeze and eczema in children. Sci Total Environ.

[5] Konno S, Hizawa N, Fukutomi Y, Taniguchi M, Kawagishi Y, Okada C (2012). The prevalence of rhinitis and its association with smoking and obesity in a nationwide survey of Japanese adults. Allergy.

[6] Kimura H, Konno S, Isada A, Maeda Y, Musashi M, Nishimura M (2015). Contrasting associations of body mass index and measles with asthma and rhinitis in young adults. Asthma Allergy Proc.

[7] Fukutomi Y, Taniguchi M, Nakamura H, Konno S, Nishimura M, Kawagishi Y (2012). Association between body mass index and asthma among Japanese adults: risk within the normal weight range. Int Arch Allergy Immunol.

[8] Taylor B, Mannino D, Brown C, Crocker D, Twum-Baah N, Holguin F (2008). Body mass index and asthma severity in the national asthma survey. Thorax.

[9] Mosen DM, Schatz M, Magid DJ, Camargo CA (2008). The relationship between obesity and asthma severity and control in adults. J Allergy Clin Immunol.

[10] Goudarzi H, Konno S, Kimura H, Makita H, Matsumoto M, Takei N (2019). Impact of abdominal visceral adiposity on adult asthma symptoms. J Allergy Clin Immunol Pract.

[11] Zerah F, Harf A, Perlemuter L, Lorino H, Lorino AM, Atlan G (1993). Effects of obesity on respiratory resistance. Chest.

[12] Bates JH (2016). Physiological mechanisms of airway hyperresponsiveness in obese asthma. Am J Respir Cell Mol Biol.

[13] Peters-Golden M, Swern A, Bird SS, Hustad CM, Grant E, Edelman JM (2006). Influence of body mass index on the response to asthma controller agents. Eur Respir J.

[14] Sutherland ER, Goleva E, Strand M, Beuther DA, Leung DY (2008). Body mass and glucocorticoid response in asthma. Am J Respir Crit Care Med.

[15] Forno E, Lescher R, Strunk R, Weiss S, Fuhlbrigge A, Celedón JC (2011). Decreased response to inhaled steroids in overweight and obese asthmatic children. J Allergy Clin Immunol.

[16] Sweeney J, Patterson CC, Menzies-Gow A, Niven RM, Mansur AH, Bucknall C (2016). Comorbidity in severe asthma requiring systemic corticosteroid therapy: cross-sectional data from the optimum patient care research database and the British thoracic difficult asthma registry. Thorax.

[17] Lugogo N, Green CL, Agada N, Zhang S, Meghdadpour S, Zhou R (2018). Obesity’s effect on asthma extends to diagnostic criteria. J Allergy Clin Immunol.

[18] Teodorescu M, Broytman O, Curran-Everett D, Sorkness RL, Crisafi G, Bleecker ER (2015). Obstructive sleep apnea risk, asthma burden, and lower airway inflammation in adults in the severe asthma research program (SARP) II. J Allergy Clin Immunol Pract.

[19] Broeckaert F, Bernard A (2000). Clara cell secretory protein (CC16): characteristics and perspectives as lung peripheral biomarker. Clin Exp Allergy.

[20] Chen LC, Zhang Z, Myers AC, Huang SK (2001). Cutting edge: altered pulmonary eosinophilic inflammation in mice deficient for Clara cell secretory 10-kDa protein. J Immunol.

[21] Hung CH, Chen LC, Zhang Z, Chowdhury B, Lee WL, Plunkett B (2004). Regulation of TH2 responses by the pulmonary Clara cell secretory 10-kd protein. J Allergy Clin Immunol.

[22] Shijubo N, Itoh Y, Yamaguchi T, Imada A, Hirasawa M, Yamada T (1999). Clara cell protein-positive epithelial cells are reduced in small airways of asthmatics. Am J Respir Crit Care Med.

[23] Taniguchi N, Konno S, Hattori T, Isada A, Shimizu K, Shimizu K (2013). The CC16 A38G polymorphism is associated with asymptomatic airway hyper-responsiveness and development of late-onset asthma. Ann Allergy Asthma Immunol.

[24] Chen LC, Tseng HM, Wu CJ, Kuo ML, Wu CJ, Gao PS (2012). Evaluation of a common variant of the gene encoding Clara cell 10 kd protein (CC10) as a candidate determinant for asthma severity and steroid responsiveness among Chinese children. J Asthma.

[25] Guerra S, Halonen M, Vasquez MM, Spangenberg A, Stern DA, Morgan WJ (2015). Relation between circulating CC16 concentrations, lung function, and development of chronic obstructive pulmonary disease across the lifespan: a prospective study. Lancet Respir Med.

[26] Burney PGJ, Luczynska C, Chinn S, Jarvis D (1994). The European community respiratory health survey. Eur Respir J.

[27] Watanabe J, Taniguchi M, Takahashi K, Nakagawa T, Ooya Y, Akazawa A (2006). Validation of ECRHS questionnaire in Japanese to use for nation-wide prevalence study of adult asthma. Arerugi.

[28] Fukui Y, Hizawa N, Takahashi D, Maeda Y, Jinushi E, Konno S (2006). Association between nonspecific airway hyperresponsiveness and Arg16Gly beta2-adrenergic receptor gene polymorphism in asymptomatic healthy Japanese subjects. Chest.

[29] Konno S, Taniguchi N, Makita H, Nakamaru Y, Shimizu K, Shijubo N (2018). Distinct phenotypes of smokers with fixed airflow limitation identified by cluster analysis of severe asthma. Ann Am Thorac Soc.

[30] Kimura H, Konno S, Nakamaru Y, Makita H, Taniguchi N, Shimizu K (2017). Sinus computed tomographic findings in adult smokers and nonsmokers with asthma analysis of clinical indices and biomarkers. Ann Am Thorac Soc.

[31] American Thoracic Society (2000). Proceedings of the ATS workshop on refractory asthma: current understanding, recommendations, and unanswered questions. Am J Respir Crit Care Med.

[32] Global initiative for asthma. https://ginasthma.org/wp-content/uploads/2021/05/GINA-Pocket-Guide-2021-V2-WMS.pdf.

[33] SAS/STAT® 14.3 user’s guide the CAUSALMED procedure. http://documentation.sas.com/?cdcId=pgmsascdc&cdcVersion=9.4_3.3&docsetId=statug&docsetTarget=statug_causalmed_syntax01.htm&locale=ja.

[34] Yung Y-F, Lamm M, Zhang W, SAS Institute Inc. Causal mediation analysis with the CAUSALMED procedure. 2018. https://www.sas.com/content/dam/SAS/support/en/sas-global-forum-proceedings/2018/1991-2018.pdf.

[35] Shijubo N, Itoh Y, Yamaguchi T, Shibuya Y, Morita Y, Hirasawa M (1997). Serum and BAL Clara cell 10 kDa protein (CC10) levels and CC10-positive bronchiolar cells are decreased in smokers. Eur Respir J.

[36] Laprise C, Boulet LP (1997). Asymptomatic airway hyperresponsiveness: a three-year follow-up. Am J Respir Crit Care Med.

[37] Xu X, Rijcken B, Schouten JP, Weiss ST (1997). Airways responsiveness and development and remission of chronic respiratory symptoms in adults. Lancet.

[38] Antó JM, Sunyer J, Basagaña X, Garcia-Esteban R, Cerveri I, de Marco R (2010). Risk factors of new-onset asthma in adults: a population-based international cohort study. Allergy.

[39] Chung KF, Wenzel SE, Brozek JL, Bush A, Castro M, Sterk PJ (2014). International ERS/ATS guidelines on definition, evaluation and treatment of severe asthma. Eur Respir J.

[40] Reynolds SD, Reynolds PR, Pryhuber GS, Finder JD, Stripp BR (2002). Secretoglobins SCGB3A1 and SCGB3A2 define secretory cell subsets in mouse and human airways. Am J Respir Crit Care Med.

[41] Guha A, Vasconcelos M, Cai Y, Yoneda M, Hinds A, Qian J, Li G (2012). Neuroepithelial body microenvironment is a niche for a distinct subset of Clara-like precursors in the developing airways. Proc Natl Acad Sci USA.

[42] Misra A, Khurana L (2011). Obesity-related non-communicable diseases: South Asians vs White Caucasians. Int J Obes.

[43] Ramachandran A, Snehalatha C (2010). Rising burden of obesity in Asia. J Obes.

[44] Hirani V, Stamatakis E (2004). Anthropometric measures, overweight, and obesity. Scott Health Surv.

