# Supplementary material for: Effects of obesity on CC16 and their potential role in overweight/obese asthma
Source: Respir Res. 2022 Jun 29;23:174. doi: 10.1186/s12931-022-02038-1 (PMC9241210; doi:10.1186/s12931-022-02038-1)
Supplement: Supplementary file 1 — Additional file 1. Table S1. Frequency of the CC16 genetic polymorphism (rs3741240). Table S2. Characteristics of non-smoking patients with lung cancer without any pulmonary diseases by BMI group. Table S3. Characteristics of the participants who attended the hospital for routine health check-ups (population 1, n = 357). Table S4. Associations between characteristics and serum CC16 levels in participants who attended the hospital for routine health check-ups (population 1, n = 357). Table S5. Characteristics of the young healthy participants (population 2, n = 137). Table S6. Association between the characteristics of young healthy participants and plasma CC16 levels (population 2, n = 137). Table S7. Characteristics of patients with asthma (population 3, n = 206). Table S8. BMI distribution in patients with severe and non-severe asthma (population 3, n = 206). Table S9. Associations between characteristics and serum CC16 levels in patients with asthma (population 3, n = 206). Fig. S1. Circulating CC16 levels based on the CC16 genetic polymorphism (rs3741240). Fig. S2. Flowchart for conducting CC16, SCBB3A2, and MUC5AC immunohistochemistry in the cancer-free lung tissues of never smoking human participants. Fig. S3. Association of circulating CC16 levels and categorical BMI in the combination of three populations excluding current smokers. Fig. S4. Decreased SCGB3A2 expressing cells in the airways of obese mice and overweight/obese humans. Fig. S5. Comparison of serum CC16 levels and blood SP-A and SP-D levels in obese vs. control mice. Fig. S6. Association of circulatory CC16 with airway hyperresponsiveness in young healthy participants (population 2). Fig. S7. Reduced serum CC16 levels among severe compared to non-severe asthma patients (population 3). Fig. S8. Inverse association of circulatory CC16 and inhaled corticosteroid (ICS) dose in asthma patients (population 3). Fig. S9. Circulatory CC16 levels according to required ICS dose in asthma patients (populatio [file 12931_2022_2038_MOESM1_ESM.pdf]

Additional file 1

Effects of obesity on CC16 and their potential role in overweight/obese asthma

Houman Goudarzi[1], Hirokazu Kimura[1], Hiroki Kimura[1], Hironi Makita[1], Munehiro Matsumoto[1], Nozomu Takei[1], Kaoruko Shimizu[1], Masaru Suzuki[1], Taku Watanabe[1], Eiki Kikuchi[1], Hiroshi Ohira[1], Ichizo Tsujino[1], Jun Sakakibara-Konishi[1], Naofumi Shinagawa[1], Noriharu Shijubo[2], Hirokazu Sato[3], Katsunori Shigehara[4,5], Kichizo Kaga[6], Yasuhiro Hida[6], Soichi Murakami[7], Yuma Ebihara[7], Akinobu Nakamura[8], Hideaki Miyoshi[9], Satoshi Hirano[7] , Nobuyuki Hizawa[10], Tatsuya Atsumi[8], Shau-ku Huang[11,12] , Yoichi M. Ito[13], Masaharu Nishimura[1], Satoshi Konno[1]

[1] Department of Respiratory Medicine, Faculty of Medicine and Graduate School of Medicine, Hokkaido University, Sapporo, Japan

[2] Department of Respiratory Medicine, JR Sapporo Hospital, Sapporo, Japan

[3] Department of Health Management, JR Sapporo Hospital, Sapporo, Japan

[4] Department of Human Immunology, Research Institute for Frontier Medicine, Sapporo Medical University School of Medicine, Sapporo, Japan

[5] Ai Medical Clinic, Sapporo, Japan

[6] Department of Cardiovascular and Thoracic Surgery, Faculty of Medicine, Hokkaido University, Sapporo, Japan

[7] Department of Gastroenterological Surgery II, Faculty of Medicine and Graduate School of Medicine, Hokkaido University, Sapporo, Japan

[8] Department of Rheumatology Endocrinology and Nephrology, Faculty of Medicine and Graduate School of Medicine, Hokkaido University, Sapporo, Japan

[9] Division of Diabetes and Obesity, Faculty of Medicine and Graduate School of Medicine, Hokkaido University, Sapporo, Japan
[10] Division of Respiratory Medicine, Institute of Clinical Medicine, University of Tsukuba, Ibaraki, Japan.
[11] National Institute of Environmental Health Sciences, National Health Research Institutes, Miaoli, Taiwan
[12] Johns Hopkins University School of Medicine, Baltimore, MD, USA
[13] Biostatics Division, Clinical Research and Medical Innovation Center, Hokkaido University Hospital, Japan

Corresponding Author: Satoshi Konno, M.D., Ph.D.
Department of Respiratory Medicine, Faculty of Medicine and Graduate School of Medicine, Hokkaido University, Sapporo, Japan
North 15 West 7, Kita-Ku, Sapporo 060-8638, Japan
E-mail: satkonno@med.hokudai.ac.jp
Fax: +81-11-706-7899; Phone: +81-11-706-5911

Materials and methods

CC16 gene polymorphism

The *CC16* A38G polymorphism (rs3741240) was identified using the TaqMan system in populations 1, 2, and 3 (assay ID, C_25473445_10; Applied Biosystems, Foster City, California) (1).

Nonspecific airway responsiveness in young healthy participants (Population 2)

Nonspecific airway responsiveness was measured by continuous methacholine inhalation with simultaneous measurement of respiratory resistance (Astograph; Chest, Tokyo, Japan), as described previously (2). After recording baseline respiratory resistance, beginning with the lowest concentration, increasing doses of methacholine were inhaled at 1-minute intervals. As an index of AHR, the cumulative dose values of inhaled methacholine measured at the inflection point at which respiratory conductance started to decrease (Dmin) were used. One Dmin unit represented a 1-minute inhalation of 1 mg/mL of methacholine (3). Logarithmic Dmin values as an index of AHR were used because previous studies have shown that AHR assessed by Dmin correlates well with degree of airway inflammation, including exhaled nitric oxide concentrations and sputum eosinophil counts, in patients with asthma (3).

Asthmatic participants (Population 3)

This study was registered in the University Hospital Medical Information Network (UMIN) Clinical Trials Registry system (UMIN ID 000003254) (https://upload.umin.ac.jp/cgi-open-bin/ctr/ctr_view.cgi?recptno=R000003917).

Smoking status

Patients were divided into three groups: current continuous smokers (Current; Affirmative question: Have you ever smoked within the last month?), former smokers who had stopped smoking for a minimum of 1 month before the initial visit (Ex; Affirmative question: Have you ever smoked within the last year?), and lifetime non-smokers (Never). We also evaluated accumulative smoking exposure by calculating pack-years (PY) through the following questions: How many cigarettes do you smoke every day? How many cigarettes had you been smoking? How many? (From ___years old to years old at per day)

Pulmonary function test

Upon study entry of population 3, spirometry was performed before and after inhalation of 400 μg oxitropium and 400 μg salbutamol. Owing to the severity of asthma in all participants, respiratory medications were not prohibited prior to the procedure, except

for the use of short-acting bronchodilators for at least 12 hours prior to all measurements. The maximum forced expiratory volume in one second (FEV_1) obtained from the two procedures and the FEV_1/forced vital capacity (FVC) values that corresponded to the maximum FEV_1 value were used for analyses. Further details of the pulmonary function tests are described in our previous report (4). For populations 1 and 2, spirometry was performed without the use of bronchodilators.

Lean vs. obese mice

C57BL/6J female mice were purchased from Japan CLEA (Tokyo, Japan). Mice were housed under a 12-h light-dark cycle and randomly divided into two groups. The first group was given ad libitum access to normal chow consisting of 25% (w/w) protein, 53% carbohydrates, 6% fat, and 8% water (Oriental Yeast Co., Ltd., Osaka, Japan), whereas the second group was given ad libitum access to a high-fat diet consisting of 25.5 (w/w) protein, 2.9% fiber, 4.0% ash, 29.4 carbohydrates, 32% fat, and 6.2% water (CLEA Japan Inc., Tokyo, Japan), both for 6 weeks. These experiments were performed twice (10 mice in experiment #1, 20 mice in experiment #2). At the end of each experiment, mouse lungs were inflated with phosphate-buffered saline, fixed in 10% formalin, and embedded in paraffin. Serum samples were also collected and stored at −80ºC until analysis. The

animal care protocol and procedures for the experiments were approved by the Animal Care Committee of Hokkaido University (17-0055).

Biomarkers in lean vs obese mice

Serum CC16, surfactant protein (SP)-A, and SP-D levels were measured using the appropriate ELISA kits (Aviva Systems Biology, San Diego, CA, USA; CUSABIO Biotech Co. Houston, TX, USA; Cloud-Clone Corp., Katy, TX, USA respectively).

RNA purification and real-time PCR for *scgb1a1* gene in lean vs obese mice

Total RNA was extracted from lung tissue from lean and obese mice with an RNeasy Mini kit (Qiagen, Hilden, Germany). The RNA was reverse transcribed with TaqMan reverse transcription reagents and reverse transcription reaction mix (Thermo Fisher Scientific) on an ABI 2720 thermal cycler (Thermo Fisher Scientific). The resulting cDNA was used as a template for real-time PCR on an ABI Prism 7300 sequence detection system (Thermo Fisher Scientific). TaqMan gene expression assay probes (Thermo Fisher Scientific) were used for mouse *scgb1a1,* which endoces CC16 protein. The β_2-microglobulin was used as the internal control.

Mouse and human lung tissue samples for immunohistochemistry

Lung tissue samples were obtained from 10 randomly selected mice (five normal control

and five obese mice). For the human samples, as shown in Fig. E2, donors included 576 non-small cell lung cancer patients who underwent lobectomy/pneumonectomy in Hokkaido University Hospital between January 2002 and December 2016, including 111 patients who were never-smokers. Of these, we excluded patients with other pulmonary diseases such as asthma and COPD using medical records, pulmonary function tests (forced expiratory volume in 1 s/forced vital capacity, flow-volume curve), and chest HRCT scan (emphysema, fibrosis). Finally, we selected patients with the lowest BMI (17.3–19.5 kg/m^2; n=10) and with the highest BMI (27.1–32.4 kg/m^2; n=9; Table E2). Mouse and cancer-free human lung samples were immunohistochemically stained for CC16, SCGB3A2, and MUC5AC using the following antibodies according to the manufacturers' instructions: anti-mouse CC10 (sc-9772; Santa Cruz Biotechnology, CA, USA), anti-human SCGB1A1 antibody (Sigma-Aldrich, St. Louis, MO), anti-mouse UGRP1/SCGB3A2 antibody (R&D Systems, Minneapolis, MN), anti-human UGRP1/SCGB3A2 antibody (R&D Systems, Minneapolis, MN), and anti-Mucin 5AC antibody [45M1] (Abcam, Cambridge, UK). The number of cells expressing each target protein in the small airways was manually and randomly counted in five randomly selected fields by two blinded examiners to determine the average percentage of positive cells/sample. This study was approved by the ethics committee of Hokkaido University

Hospital (017-0258).

Covariates and statistical analysis

For the assessment of the association between BMI and circulatory CC16 levels, we created three adjusted models. Model 1 (partially adjusted model) was adjusted by age, sex, smoking status, and history of doctor-diagnosed asthma for Population 1, adjusted by sex, age, and smoking status for Population 2, adjusted by age, sex, smoking status, and asthma severity for Population 3, and adjusted by age, sex, and smoking status for the combination of Populations 1, 2, and 3. Model 2 (fully adjusted model) was adjusted for covariates in Model 1 and the *CC16* A38G polymorphism (rs3741240). Lastly, we excluded current smokers to minimize the influence of smoking on the association between BMI and CC16 levels.

References

S1. Taniguchi N, Konno S, Hattori T, et. al. The CC16 A38G polymorphism is associated with asymptomatic airway hyper-responsiveness and development of late-onset asthma. Ann Allergy Asthma Immunol. 2013;111(5):376-381.e1

S2. Fukui Y, Hizawa N, Takahashi D, et al. Association between nonspecific airway hyperresponsiveness and Arg16Gly beta2-adrenergic receptor gene polymorphism in asymptomatic healthy Japanese subjects. Chest. 2006;130:449e454.

S3. Niimi A, Matsumoto H, Takemura M, Ueda T, Chin K, Mishima M. Relationship of airway wall thickness to airway sensitivity and airway reactivity in asthma. Am J Respir Crit Care Med. 2003;168:983e988.

S4. Kimura H, Konno S, Nakamaru Y, et al. Sinus Computed Tomographic Findings in Adult Smokers and Nonsmokers with Asthma Analysis of Clinical Indices and Biomarkers. Ann Am Thorac Soc. 2017;14(3):332-341.

Table S1. Frequency of the *CC16* genetic polymorphism (rs3741240).

CC16 (A38G) genotype	n	CC16 geometric mean (95% CI), ng/mL	p for trend
Population 1			
AA	45	5.0 (4.5-5.6)	
AG	126	6.1 (5.7-6.5)	
GG	105	6.6 (6.2-7.2)	<0.0001
Population 2			
AA	25	5.2 (4.4-6.3)	
AG	61	5.9 (5.1-6.7)	
GG	51	6.9 (6.0-7.8)	0.024
Population 3			
AA	42	5.6 (4.5-6.9)	
AG	89	7.0 (6.2-7.8)	
GG	75	7.5 (6.5-8.7)	0.010

Table S2. Characteristics of non-smoking patients with lung cancer without any pulmonary diseases by BMI group.

Participants with low BMI (BMI less than 19.5 kg/m^2)						
No.	BMI	Sex	Age	VC (L)	FEV_1 (L)	FEV_1/FVC (%)
1	18.0	M	76	2.73	2.08	81.9
2	18.2	F	44	2.99	2.85	96.6
3	17.3	F	64	2.77	2.17	82.2
4	17.5	F	52	3.79	3.14	85.3
5	17.9	F	65	2.92	2.47	90.2
6	14.6	F	59	2.92	2.4	81.9
7	18.4	F	55	2.47	2.29	80.9
8	18.5	F	63	2.88	2.37	81.5
9	19.0	F	81	2.32	1.69	73.5
10	19.2	F	73	3.05	2.15	73.9
Participants with high BMI (BMI less than 27 kg/m^2)						
No.	BMI	Sex	Age	VC (L)	FEV_1 (L)	FEV_1/FVC (%)
1	30.1	F	61	2.51	2.04	82.3
2	32.4	F	77	2.05	1.56	83.4
3	30.1	F	79	2.75	1.93	72.6
4	27.8	F	69	2.10	1.61	74.1
5	27.5	F	74	2.45	1.99	82.2
6	27.3	M	77	3.62	2.77	78.9
7	28.6	F	75	2.39	1.76	71.8
8	27.1	F	70	2.36	1.89	80.6
9	27.5	F	75	3.08	2.34	79.7

Table S3. Characteristics of the participants who attended the hospital for routine health check-ups (population 1, n = 357).

	n (%), or mean ± SD
Female, n (%)	154 (43.1)
BMI (kg/m^2)	22.7 ± 3.1
BMI category	
<18.5	21 (5.9)
≥18.5, <25	261 (73.1)
≥25	75 (21.0)
Smoking status (never/ex-smoker/current), n	170 (47.6)/ 87 (24.4)/ 100 (28.0)
Age, years	
≥20-<30	3 (0.8)
≥30-<40	32 (9.0)
≥40-<50	107 (30.0)
≥50-<60	183 (51.2)
≥60	32 (9.0)
History of doctor-diagnosed asthma, n (%)	49 (13.9)
*Current wheeze, n (%)	33 (9.3)
Pulmonary function tests	
VC (L)	3.6 ± 0.8
%VC	113.1 ± 13.9
FEV_1 (L)	2.9 ± 0.6
FEV_1, predicted %	105.1 ± 13.7
FEV_1/FVC, %	82.4 ± 6.9
< 70%	12 (3.4)
≥ 70%	336 (96.6)

* The European Community Respiratory Health Survey (ECRHS) questionnaire

Table S4. Associations between characteristics and serum CC16 levels in participants who attended the hospital for routine health check-ups (population 1, n = 357).

	serum CC16 concentration (ng/mL)	p-value
Sex		
Male	6.5 (6.2-6.9)	
Female	5.6 (5.3-6.0)	<0.001
Smoking status		
never	6.4 (6.0-6.7)	
ex-smoker	6.5 (6.0-7.0)	
current	5.5 (5.0-6.0)	0.003
Age (years)		
≥20-<30	8.0 (1.5-42.6)	
≥30-<40	5.8 (5.0-6.7)	
≥40-<50	6.1 (5.7-6.6)	
≥50-<60	6.0 (5.7-6.4)	
≥60	6.8 (6.0-7.7)	0.343
History of doctor-diagnosed asthma		
yes	6.2 (5.5-7.0)	
no	6.1 (5.8-6.4)	0.816
*Current wheeze		
yes	6.0 (5.2-7.0)	
no	6.1 (5.9-6.4)	0.807
FEV_1/FVC		
FEV_1/FVC < 70% (n = 12)	5.8 (4.7-7.1)	
FEV_1/FVC ≥ 70% (n = 336)	6.1 (5.9-6.4)	0.602

Data are geometric means (95% CI) or Spearman's correlation coefficients (ρ) for the correlation with CC16 levels.

* The European Community Respiratory Health Survey (ECRHS) questionnaire

Table S5. Characteristics of the young healthy participants (population 2, n = 137)

	n (%), or mean ± SD
Age, yr	24.0 ± 3.4
Female, n (%)	40 (29.2)
BMI (kg/m^2)	21.6 ± 2.6
BMI category (kg/m^2)	
<18.5	6 (4.4)
≥18.5, <25	120 (87.6)
≥25	11 (8.0)
Smoking status (never/ex-smoker/current)	115 (83.9)/ 8 (5.9)/ 14 (10.2)

Table S6. Association between the characteristics of young healthy participants and plasma CC16 levels (population 2, n = 137).

	Plasma CC16 concentration (ng/mL)	p-value
Sex		
Male	6.0 (5.4-6.6)	
Female	6.4 (5.4-7.6)	0.461
Smoking status		
never	6.3 (5.7-6.8)	
ex-smoker	4.1 (2.2-7.5)	
current	6.2 (5.0-7.7)	0.056

Plasma CC16 concentration is shown in geometric means (95% CI).

Table S7. Characteristics of patients with asthma (population 3, n = 206).

	Total (n = 206)	Non-severe asthma (n = 79)	Severe asthma (N = 127)	p-value
Age, yr[a]	59.4 ± 13.8	61.8 ± 14.7	57.9 ± 13.0	**0.048**
Female, n (%)[a]	123 (59.7)	47 (59.5)	76 (59.8)	0.960
Age of asthma onset, yr[a]	39.7 ± 18.3	42.3 ± 19.3	38.2 ± 17.6	0.119
Asthma duration, yr[a]	19.6 ± 15.1	19.5 ± 15.6	19.7 ± 14.8	0.928
BMI (kg/m^2)[a]	24.9 ± 5.0	23.9 ± 4.0	25.6 ± 5.4	**0.020**
Blood neutrophils (10^9/L)[c]	3.9 (3.6-4.1)	3.0 (2.8-3.3)	4.5 (4.2-4.8)	**<0.001**
Blood eosinophils (cells/μl)[c]	214.5 (186.6-246.6)	233.7 (198.3-275.5)	203.4 (166.1-249.1)	0.340
Total serum IgE (IU/mL)[c]	166.0 (135.3-203.6)	189.3 (141.6-253.1)	153.2 (115.8-202.6)	0.323
Atopy, yes[a]	140 (68.0)	59 (74.7)	81 (63.7)	0.099
FeNO (p.p.b)[c]	29.1 (26.1-32.5)	26.1 (22.2-30.7)	31.2 (26.9-36.1)	0.117
Pack-years[b]	66.0 (0-376.2)	20.0 (0-231.0)	100.0 (0-465.0)	0.170
Smoking (never/ex-/current), n	21/ 97/ 88	8 / 35 / 36	13 / 62 / 52	
FEV_1 (%predicted) [a]	87.0 ± 20.0	93.8 ± 19.1	82.7 ± 19.4	**<0.001**
FEV_1 (L) [a]	2.14 ± 0.72	2.27 ± 0.68	2.06 ± 0.73	**0.037**
FEV_1/FVC (%)[a]	65.3 ± 12.8	66.9 ± 12.4	64.3 ± 13.0	0.157
*ICS dose, μg[a]	1208.4 ± 731.7	500.9 ± 228.4	1648.6 ± 574.3	**<0.001**
OCS use, yes[a]	45 (21.8)	0 (0)	45 (35.4)	**<0.001**

[a] Mean ± SD, [b] median (IQR), [c] geometric mean (95% lower and upper mean).

*Equivalent to budesonide dose.

Table S8. BMI distribution in patients with severe and non-severe asthma (population 3, n = 206).

BMI (kg/m^2)	N	Total	Non-severe asthma (n = 79)	Severe asthma (N = 127)	p-value
Continuous	206	24.9 ± 5.0	23.9 ± 4.0	25.6 ± 5.4	0.020[a]
Categorical					
<18.5	11	11 (5.3)	5 (6.3)	6 (4.7)	-
18.5 ≤ BMI <25	105	105 (50.1)	48 (60.8)	57 (44.9)	-
BMI ≥ 25	90	90 (43.7)	26 (32.9)	64 (50.4)	0.023[b]

Values are sample numbers (%) for the categorical classification.

[a] ANOVA, [b] Chi-square.

Table S9. Associations between characteristics and serum CC16 levels in patients with asthma (population 3, n = 206).

	serum CC16 concentration (ng/mL)	p-value
Sex		
Male	7.7 (6.9-8.7)	-
Female	6.3 (5.6-7.1)	0.016
Smoking status		
Current	6.6 (5.2-8.3)	-
Non-current (never, and ex-smoker)	6.9 (6.3-7.5)	0.766
Pack-years		
PY <10	6.6 (5.9-7.3)	
PY ≥10	7.3 (6.5-8.3)	0.202
Age (years)		
<40 (n=23)	5.7 (4.4-7.4)	-
≥40-<50 (n=25)	4.7 (3.5-6.3)	-
≥50-<60 (n=43)	5.8 (4.8-7.0)	-
≥60-<70 (n=58)	7.4 (6.5-8.4)	-
≥70 (n=57)	9.1 (7.9-10.5)	<0.0001
Asthma severity		
mild/moderate	8.2 (7.2-9.4)	-
Severe	6.1 (5.5-6.8)	0.0006

Data are geometric means (95% CI) or Spearman's correlation coefficients (ρ) for the correlation with CC16 levels.

Fig. S1. Circulating CC16 levels based on the *CC16* genetic polymorphism (rs3741240).

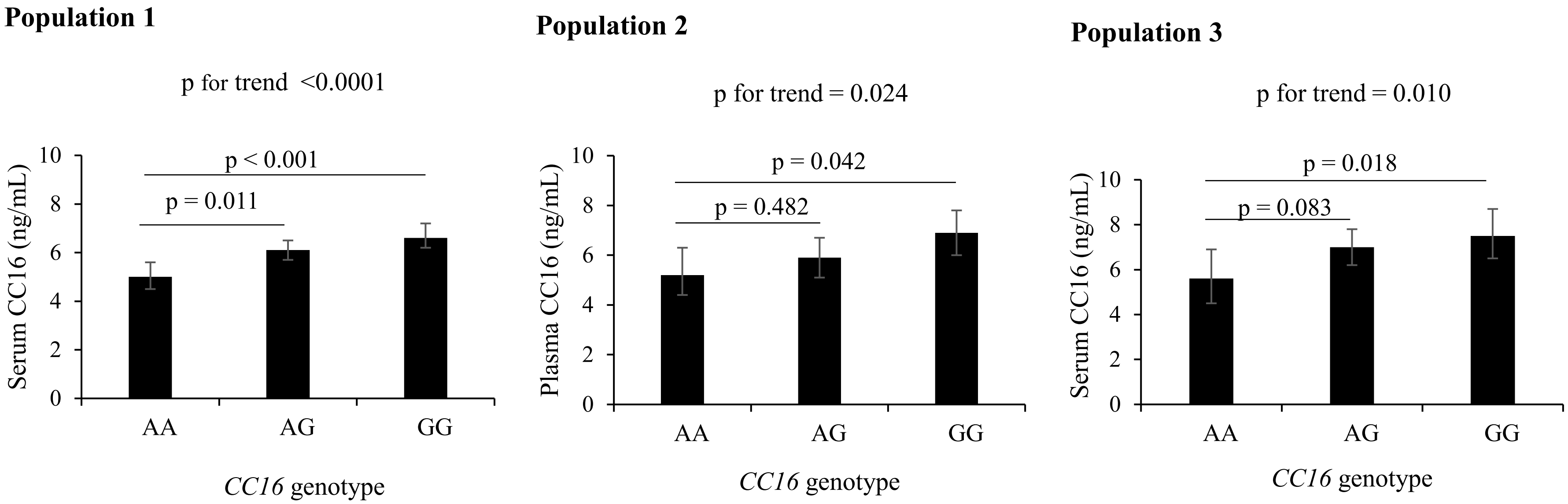

CC16 levels are shown as the geometric mean (95% CI).

Fig S2. Flowchart for conducting CC16, SCBB3A2, and MUC5AC immunohistochemistry in the cancer-free lung tissues of never smoking human participants.

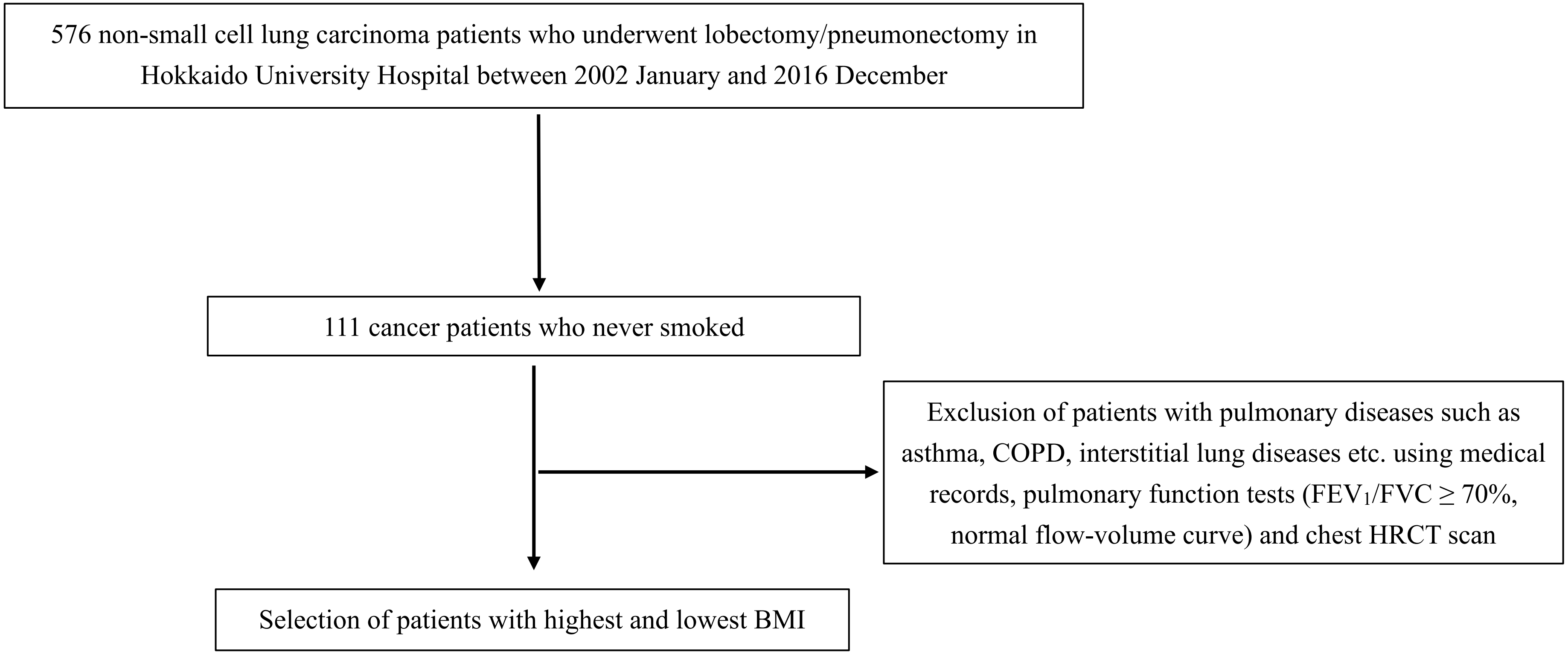

Fig S3. Association of circulating CC16 levels and categorical BMI in the combination of three populations excluding current smokers.

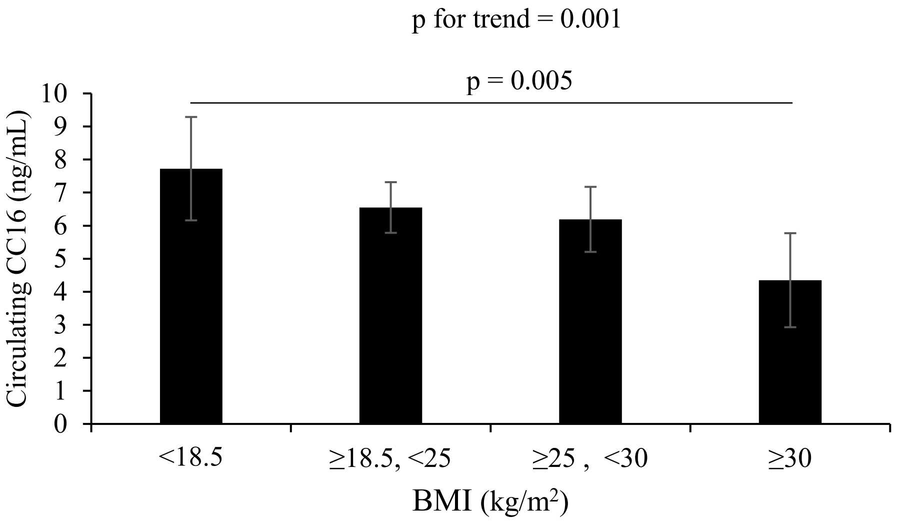

	P for trend
Crude	0.014
Model 1 (partially adjusted)	0.001
Model 2 (fully adjusted)	0.001

The values are shown as the least-square means, and the error bars depict the upper and lower 95% CI. Figures indicate fully adjusted associations between BMI values and circulatory CC16 levels (ng/mL). Model 1 (partially adjusted model): adjusted by age, sex. Model 2 (fully adjusted model): adjusted for covariates in Model 1 and the *CC16* A38G polymorphism (rs3741240).

Fig S4. Decreased SCGB3A2 expressing cells in the airways of obese mice and overweight/obese humans.

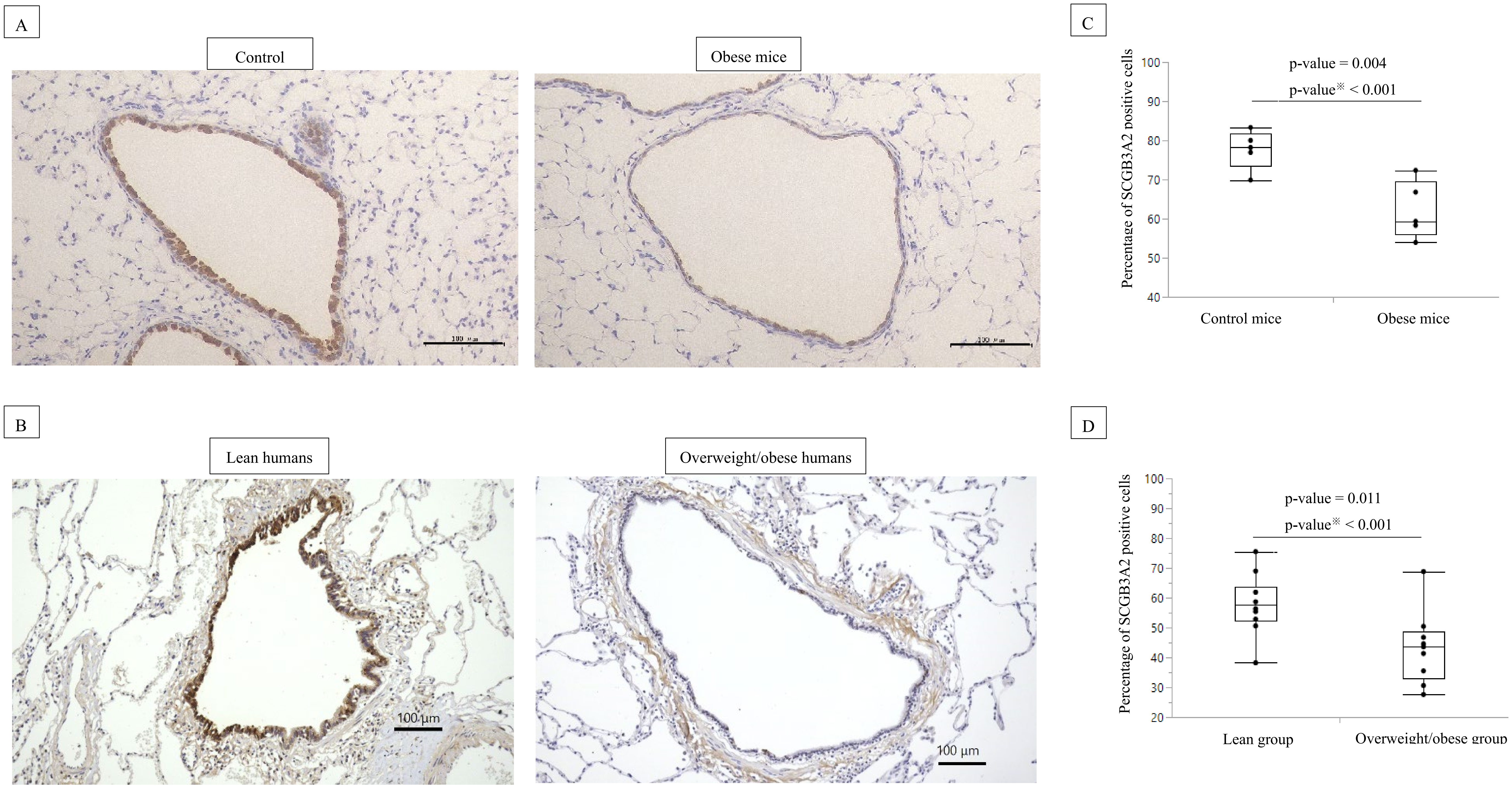

Representative photomicrographs of SCGB3A2 immuno-histochemical staining of small airways from obese vs. control mice (n = 5 mice per group) (A), and small airways from overweight/obese vs. lean humans (B). Comparison of the percentage of SCGB3A2 expressing cells in the small airways of obese vs. normal control mice (n = 5 mice per group) (C). Comparison of the percentage of SCGB3A2 expressing cell in the small airways of overweight/obese vs. lean humans (n = 10 for the lean group, and 9 for the overweight/obese group) (D). The scale bars are 100 µm. ※split-plot method (ANOVA)

Fig. S5. Comparison of serum CC16 levels and blood SP-A and SP-D levels in obese vs. control mice.

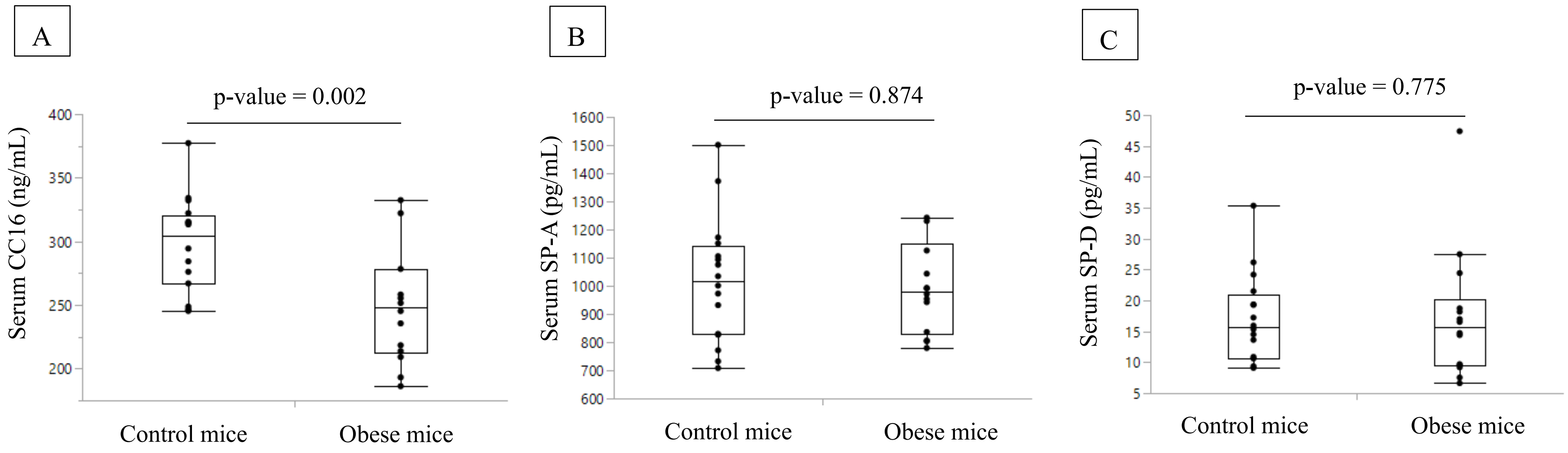

n = 16 mice for the control group and n = 14 mice for the obese mice group.

Fig. S6. Association of circulatory CC16 with airway hyperresponsiveness in young healthy participants (population 2).

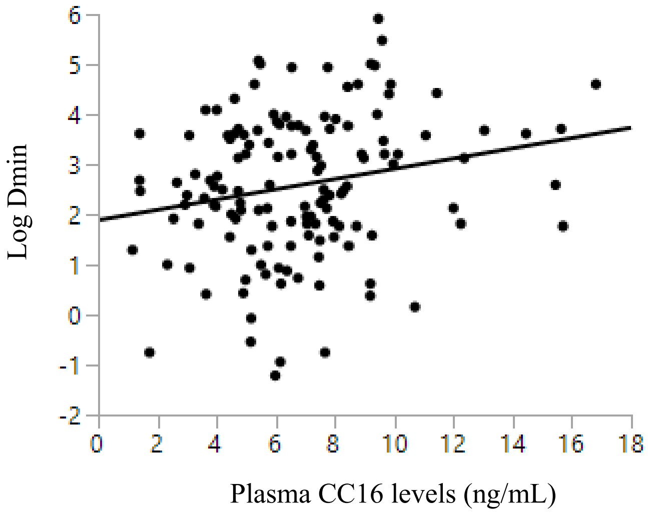

	Estimates (95% CI)	p-value
Crude	0.102 (0.023-0.182)	0.011
Model 1 (age, sex, smoking, *CC16* polymorphism)	0.095 (0.013-0.178)	0.023
Model 2 (covariates in Model 1 and BMI)	0.085 (0.001-0.169)	0.045

The figure indicates the unadjusted association between serum CC16 levels and Dmin (log). Airway hyperresponsiveness was assessed by the logarithmic cumulative dose of inhaled methacholine measured at the inflection point at which respiratory conductance starts to decrease (log Dmin).

Fig. S7. Reduced serum CC16 levels among severe compared to non-severe asthma patients (population 3).

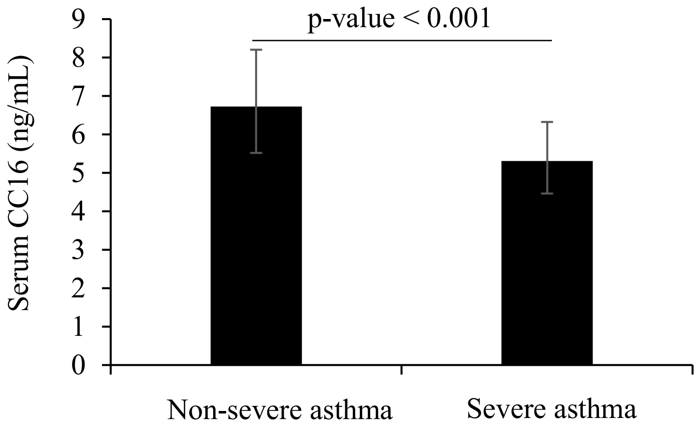

	p-value
Crude	< 0.001
Model 1 (age, sex, smoking status)	0.001
Model 2 (covariates in Model 1 and *CC16* polymorphism)	0.003
Model 3 (covariates in Model 2 and BMI)	0.009

The values in B are shown in the least square means (95% CI). The figure shows the unadjusted association between asthma severity and circulatory CC16 levels (ng/mL).

Fig. S8. Inverse association of circulatory CC16 and inhaled corticosteroid (ICS) dose in asthma patients (population 3).

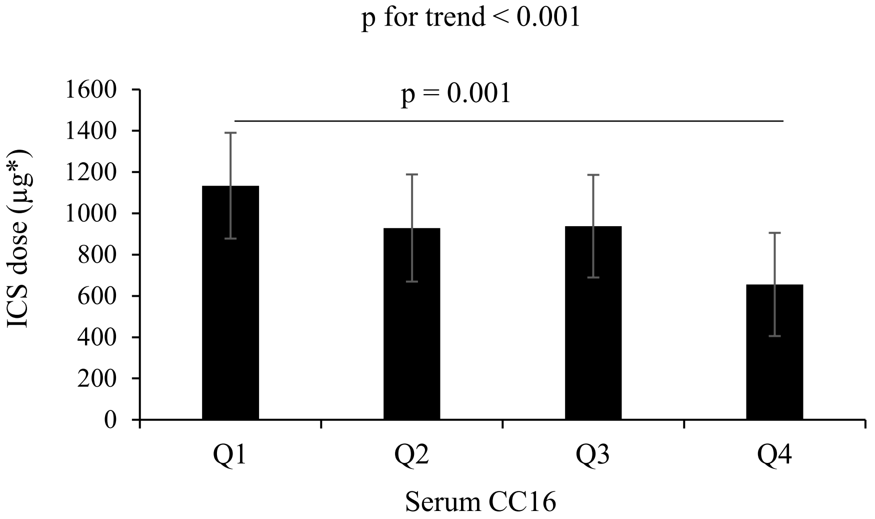

	p for trend
Crude	<0.001
Model 1 (age, sex, smoking status, pack-years, *CC16* polymorphism)	0.002
Model 2 (covariates in Model 1 and BMI)	0.013

The values are shown with the least square means (± standard errors). Patients with current oral corticosteroid use were excluded (n = 45). The figure indicates unadjusted associations between values of serum CC16 and ICS dose. *Equivalent to budesonide dose. Q: Quartile.

Fig S9. Circulatory CC16 levels according to required ICS dose in asthma patients (population 3).

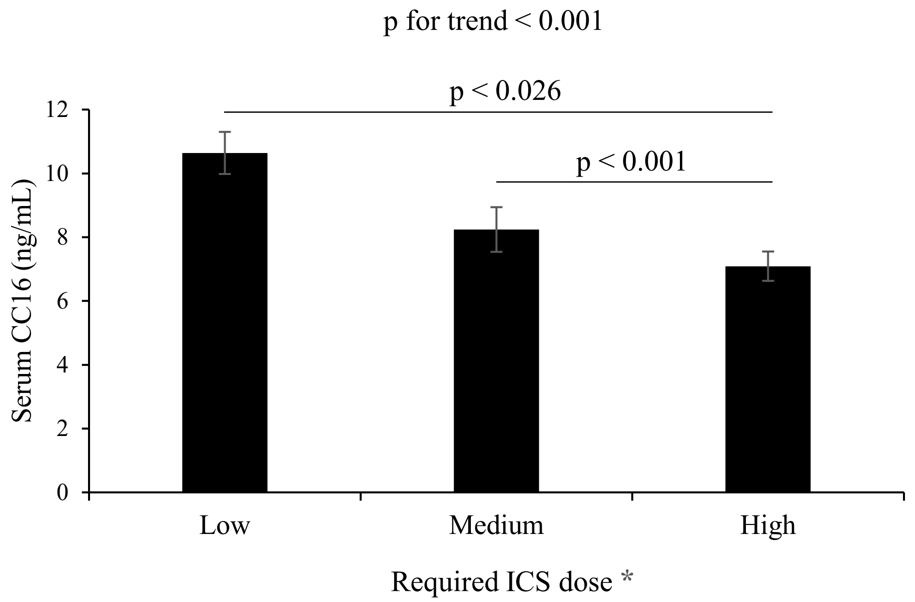

	p for trend
Crude	<0.001
Model 1 (age, sex, smoking status, pack-years, *CC16* polymorphism)	<0.001
Model 2 (covariates in Model 1 and BMI)	0.001

The values are shown with the least square means (± standard errors). Patients with current oral corticosteroid use were excluded (n = 45). The figure indicates unadjusted associations between values of serum CC16 and ICS dose.

*Equivalent to budesonide dose and divided to low (n=42), medium (n=37), and high dose groups (n=82) according to the cutoff values provided by GINA guideline.
